# Sleep Duration, Number of Awakenings and Arterial Stiffness in Industrial Shift Workers: A Five-Week Follow-Up Study

**DOI:** 10.3390/ijerph19041964

**Published:** 2022-02-10

**Authors:** Dagfinn Matre, Per Anton Sirnes, Elisabeth Goffeng, Øivind Skare, Marit Skogstad

**Affiliations:** 1 Division of Research, National Institute of Occupational Health, 0636 Oslo, Norway; elisabeth.goffeng@stami.no (E.G.); oivind.skare@stami.no (Ø.S.); marit.skogstad@stami.no (M.S.); 2 Østlandske Hjertesenter, 1523 Moss, Norway; pas@cardio.no

**Keywords:** shift work, night work, cardiovascular diseases, occupational health, sleep, sleep duration, hypertension

## Abstract

Shift work may increase the risk for hypertension and arterial stiffness, potentially a consequence of disturbed sleep. The aim of this study was to investigate possible correlations between sleep length and spontaneous awakenings with selected cardiovascular risk factors in shift workers at an industrial plant. We examined 19 shift workers by means of blood pressure and arterial stiffness, measured as pulse wave velocity (PWV), prior to and after a 5-week shift period. Sleep patterns were monitored on a daily basis with the assistance of a smartphone-based sleep diary (the entire test period) and by actigraphy (limited to 2 weeks). The number of awakenings and total sleep time were calculated. Shorter sleep duration was associated with higher blood pressure and partly with higher PWV, indicating an increased risk of cardiovascular disease (CVD) with reduced sleep duration. Unexpectedly, a lower number of awakenings was associated with an increase in blood pressure, indicating a reduced risk of CVD. No other significant associations were determined. The results from the present study among shift workers in Norway could support the hypothesis that short sleep duration is associated with elevated blood pressure and arterial stiffness.

## 1. Introduction

Recent trends show a rapid increase in shift work and flexible schedules, but there is a shortage of studies evaluating the potential health effects in relation to this type of work schedule. In Europe 21% of the workforce is engaged in shift work, and 19% work at night [1].

Cardiovascular disease (CVD) is related to approximately one-third of deaths worldwide [2]. Hence, identifying modifiable risk factors for CVD is of great importance. A systematic review and meta-analysis reported an association between shift work and CVD [3]. It has been estimated that the risk of CVD, after an initial period of 5 years, increases by 7.1% for every subsequent 5 years of cumulative shift work exposure [4]. Shift workers are vulnerable to sleep disturbances/sleep disorders, predominantly among those working night shifts and early morning shifts [5]. Extended working hours can also be associated with excessive sleepiness [6] and lack of sleep [7].

Short sleep duration, or awakenings after sleep onset, has been associated with an increased risk of CVD [8]. Short sleep duration has also been associated with a higher risk of hypertension [9] and an increase in blood pressure (BP) among shift workers [10]. Keeping a sleep diary is a basic tool in insomnia research [11]. The present study used a consensus sleep diary [12] in combination with actigraphy. Activity-based sleep-wake monitoring or actigraphy is a key assessment tool in sleep medicine, but actigraphy should be complemented by a sleep diary [13].

Several mechanisms may account for an elevated CVD risk among shift workers These mechanisms include endocrinological disturbances [14], increased systemic inflammation [15,16], change to the immune system [17], or circadian disruption [18]. The detection of early manifestation of arterial stiffness is important to reduce the patients’ risk of CVD. Pulse wave velocity (PWV) is a measure of arterial stiffness and an independent predictor of cardiovascular disease and all-cause mortality in the general population [19]. Arterial stiffness is one of the earliest manifestations of vascular damage [20] and could coincide with frequent awakenings from sleep [21]. 

In a 3-year prospective study of shift workers in two industrial plants in Norway, we analyzed blood parameters, brachial blood pressure, resting heart rate and central blood pressure, augmentation pressure and index, and pulse wave velocity. We performed ultrasound measurement of the carotid arteries, and measured VO_2max_ [22]. We found an association between shift work and risk factors for cardiovascular disease such as increased carotid intima media thickness and elevated C-reactive protein (CRP) [23]. The current study differed from many others due to the combination of day-to-day measurements of sleep, capturing acute sleep debt, and cardiac stiffness in a real-life situation. 

The aim of the current study was to examine the possible inter-individual associations between sleep duration, number of awakenings and blood pressure and arterial stiffness in industrial shift workers. 

## 2. Materials and Methods

The current study was part of a 3-year prospective follow-up study in relation to cardiovascular health effects of shift work, night shifts and long working hours among industrial workers (see published protocol [22]). Initially, 94 participants were recruited from two industrial plants in Norway. According to the protocol article preceding the current study [22], participants with severe cardiovascular and lung disease were excluded from the study. Two additional exclusion criteria were cancer and blood pressure exceeding 180/110 mmHg. In the current study, workers were invited to participate in a detailed follow-up study of sleep patterns over a 5-week shift period, including two examinations involving PWV and CVD risk factors. The participants worked rotating day, evening and night shifts lasting for 8 or 12 h. The participants worked on adjacent shifts and those with a high degree of compliance in a former study [24] were asked to participate. Nineteen of 30 eligible workers (two women) consented to participate. For a more detailed shift plan, see [23]. The examinations/tests took place in August 2019, after the participants had returned from their holiday. To minimize interference with the circadian rhythm, the participants were assessed for a minimum of 3 days and nights following the last preceding night shift. After the 5-week shift period, a second assessment was carried out following the same protocol. Data from the second assessment were used in the regression analysis. 

Following 5 minutes of seated rest, blood pressure and resting heart rate in the left arm was measured 3 times at 1-min intervals by BpTRU^®^ (Bp TRU medical devices, Coquitlam, BC, Canada). The average of three measurements of the seated systolic blood pressure (ssBP) and the seated diastolic blood pressure (sdBP) was calculated. Pulse wave velocity (PWV) was assessed by SphygmoCor XCEL^®^ (AtCor Medical Pty Ltd., Sydney, Australia), according to the manufacturer’s recommendations, using the average result of the three measurements. 

Sleep patterns were monitored using wrist-worn actigraphy (AX3, Axivity Ltd., Newcastle upon Tyne, UK) during a 2-week period and by a smartphone-based sleep diary derived from the Consensus Sleep Diary-Core for the duration of 5 weeks [12]. At 21.00 each evening, the participants received a text message, and by clicking on a URL, they accessed a web browser with questions concerning time going to sleep, sleep latency, time of final awakening, number of awakenings (NA), and wakefulness after sleep onset the night prior. Sleep duration was measured as total sleep time (TST) and calculated by subtracting sleep onset latency and wakefulness after sleep onset from the difference between the time going to sleep and the time of final awakening [25]. Similarly, an objectively based measurement was calculated by the use of actigraphy [26] for 2 weeks of the 5-week shift period. 

Linear regression analyses were used to determine whether TST or NA were associated with the blood pressure and PWV measurements obtained following the 5-week shift period, using a between-subjects design. Systolic blood pressure, diastolic blood pressure and PWV were treated as dependent variables in separate analyses, one for each sleep variable (NA and TST assessed by diary or by actigraphy). The results are presented as crude models and as models adjusted for age and sex. Comparison of reliability in relation to NA and TST assessments was performed by intraclass correlation coefficient (ICC). All analyses were carried out using Stata v.16.1 (StataCorp LLC, College Station, TX, USA). The threshold for significance was α = 0.05. 

## 3. Results

Table 1 illustrates the characteristics of the shift workers; age, body-mass index (BMI) and years working shifts, as well as blood pressure and PWV results from the examination prior to and following the 5-week shift period. The measured parameters were quite stable across the 5-week shift period. 

### 3.1. Sleep Measurements from Diary and Actigraphy

The sleep diary was completed for 25.1 ± 7.9 (mean ± standard deviation) of 35 days (72%). Valid actigraphy data were available for 10.0 ± 2.5 of 14 days (71%). Subjects reported significantly fewer awakenings (NA) in the sleep diary, compared to NA measured by actigraphy (Table 2). Longer sleep duration (TST) was reported by diary, compared to TST measured by actigraphy (Table 2). The reliability comparison between diary and actigraphy measurements was ICC = 0.50 and ICC = 0.33 for TST and NA, respectively. 

### 3.2. Association between Sleep Measurements and Cardiovascular Risk Factors

**Figure 1:**
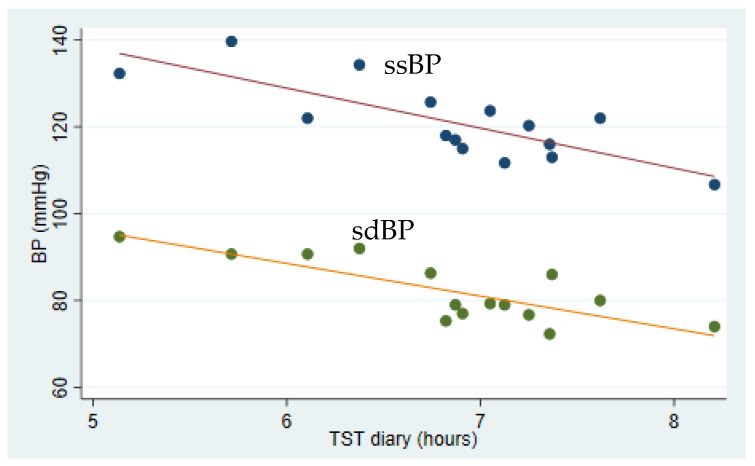
Association between average sleep duration and blood pressure among industrial shift workers. Sleep was measured as total sleep time by diary (TST diary) for 5 weeks preceding measurements of systolic and diastolic blood pressure (BP). A negative association between both systolic and diastolic blood pressure, and total sleep time in the adjusted analyses was identified (*p* = 0.005, R2 = 0.64 and *p* = 0.003, R2 = 0.64, respectively). Solid lines show linear prediction.

**Figure 2:**
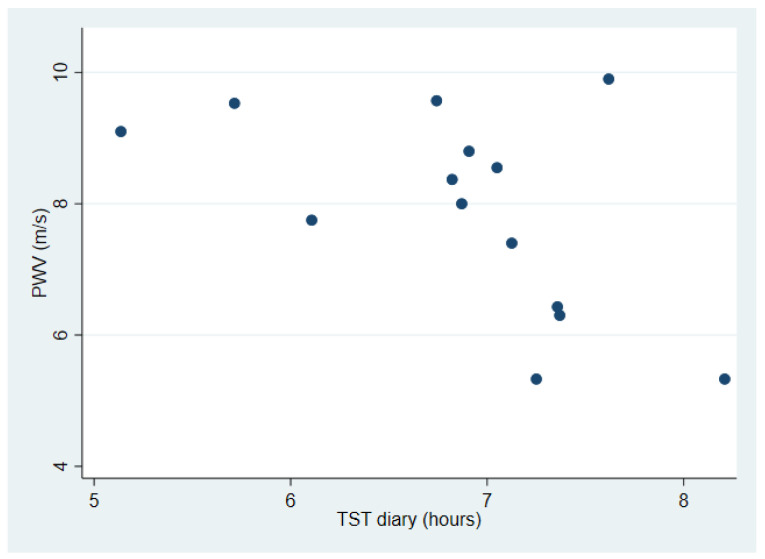
Association between average sleep duration and pulse wave velocity (PWV) among industrial shift workers. Sleep was measured as total sleep time by diary (TST diary) for 5 weeks preceding measurements of systolic and diastolic blood pressure (BP).

Table 3 shows the associations between sleep measurements, blood pressure and pulse wave velocity. A negative association between TST measured by diary and ssBP was identified, both in the crude (*p* < 0.001) and in the adjusted analysis (*p* = 0.005) (Figure 1). Similar associations were found between TST measured by/registered in diary and sdBP, both in the crude (*p* < 0.001) and adjusted analysis (*p* = 0.003). Furthermore, a negative association between TST registered in diary and PWV (*p* = 0.045) was identified, but limited to the crude analysis (Figure 2). A post hoc analysis showed a significant bivariate correlation between age and PWV (rho = 0.70, *p* = 0.0039). A negative association between NA measured by actigraphy and ssBP in the crude analysis (*p* = 0.031), and between NA and sdBP both in the crude (*p* = 0.001) and adjusted analysis (*p* = 0.008) was identified. NA measured by diary was not associated with blood pressure, nor was NA associated with PWV.

## 4. Discussion

The results from the present study among rotating shift workers in industry suggest that shorter sleep duration, as measured by sleep diary, was associated with elevated blood pressure and arterial stiffness, reported by partly increased PWV, indicating an elevated CVD risk. Unexpectedly, more frequent number of awakenings after sleep onset was associated with lower blood pressure, indicating a reduced CVD risk. 

The negative association between sleep duration and blood pressure is consistent with findings in several studies. A population-based prospective study including more than 9000 participants suggests that short sleep duration among shift workers could indicate a risk for hypertension [10]. That short sleep duration increases the risk for hypertension is also supported by a study among adults older than 40 years [27], and by a study among 578 Americans of similar BMI and age as the participants in the present study [28]. The regression coefficient was considerably smaller in the latter study (systolic BP −1.80 mmHg per hour sleep in the adjusted model), when compared to the present/current study (systolic BP −8.78 mmHg per hour sleep). This inconsistency may be explained by the limited number of participants in the current study, resulting in a greater degree of variation in blood pressure measurements. The findings of an additional American study, however, were limited to an association between sleep duration and blood pressure in African-American shift workers [29]. Other studies with contrasting findings are also available. A study among non-insomniac elderly subjects (72 ± 1 years) failed to identify an association between sleep duration, measured by the Pittsburg sleep quality index questionnaire, and hypertension [30]. The lack of association could be the result of the age range, relative to the present population. 

We have previously shown a decrease in blood pressure following an 8-week physical activity initiative focusing on cardiorespiratory performance in the same group of workers [24], but these results may be temporary depending on the level of future physical activity. In the present study, self-reported short sleep duration was associated with elevated blood pressure inter-individually in a relatively small population of workers. 

The present data partially support the perception that short sleep duration may be associated with arterial stiffness, measured as elevated PWV. However, the association was not to be found in the adjusted analysis (Table 3), suggesting age as a key confounder in the association between sleep duration and PWV. A post hoc analysis confirmed that age and PWV were positively correlated. That fact that PWV increases with age has been previously established [31]. However, other studies have shown that long-term exposure to night shift work [32] or shift work [33] seems to be associated with arterial stiffness, measured by pulse wave velocity (PWV). Comparing clockwise and counterclockwise shift work rotations failed to reveal any differences in PWV among male steel factory workers [34], a population quite comparable to the participants in the current study. As the present small number of participants exhibited a noticeable variance in sleep duration (Figure 1), the difference may be too insignificant to affect PWV, taking the age range into account. The above-mentioned studies, by Chen et al. [33] in particular, indicate that changes in PWV take years to develop. However, PWV values may, generally, be reduced as a result of weight loss, regular exercise, reduced salt intake and calorie restrictions [35]. Blood pressure treatment, statins and smoking cessation will also contribute to a reduction of arterial stiffness [20]. Interestingly, an association between shift work and CRP at baseline was found [23], which may be linked to sleep disturbances within the group. Sleep deprivation can cause an upregulation of genes controlling inflammation and thus CRP [17]. To further study the association between arterial stiffness, inflammation, PWV and shift work, a prospective design with long follow-up is necessary.

Frequent awakenings are one of the key elements in insomnia, and insomnia is a risk factor for hypertension [36]. The negative association between NA, assessed by actigraphy, and blood pressure suggests that a higher number of awakenings reduces blood pressure. This is a surprising finding. A possible explanation could be that the algorithm calculating the number of awakenings was too sensitive. This explanation is somewhat supported by the findings reported in Table 2, indicating that NA measured by actigraphy was tenfold compared to NA registered in the diary. Both sleep variables estimated by actigraphy differed significantly from those measured by the smartphone sleep diary. The actigraphy-based estimates of NA and TST were all within the range of those reported in a recent study comparing different algorithms [37]. In insomniacs, estimated TSTs are typically shorter if measured by diary than by polysomnography [38], contrary to the findings in the current study. Estimated TSTs were longer in diary registrations than those measured by actigraphy. Hence, the subjects may have a subjective experience of sleeping quite well. Offshore night shift workers have reported longer TSTs by diary than by actigraphy [39]. This may indicate a difference between insomniacs and regular shift workers. As actigraphy may register short sleep alterations not perceived as real awakenings by the subject, the actigraphy-based estimates of awakening may be more accurate. The significant difference between diary-based and actigraphy-based sleep measurements complicates the interpretation of the associations between sleep and risk of CVD. 

The diary design of the current study represented a strength. It was likely to reduce recall bias, as participants were asked to recall sleep daily. Accurate sleep measures were then linked to the follow-up measurement of blood pressure and PWV. Another strength is that the same technicians performed all tests, providing the participants with identical guidelines in every session. The fact that the AX3 actigraphy algorithm calculating sleep duration [26] had not been validated by polysomnography for non-patient subjects, or for daytime sleepers, represents a limitation. A further limitation was the relatively low compliance between diary and actigraphy measurements (around 70%), as actigraphy measurements did not correspond exactly with diary measurements. Albeit a small group, the participants were all rotating shift workers selected as a result of high compliance in a previous sub-study [24]; consequently, the selection of healthy and highly motivated individuals into the study should be taken into consideration. 

## 5. Conclusions

The results from the present study among a Norwegian population of industrial shift workers support the hypothesis that short sleep duration and number of awakenings are associated with an increase in blood pressure and possibly arterial stiffness. The current study had a relatively small sample size, and the findings may be somewhat limited to be considered representative of a population. To further study the relevant associations, a prospective design study with long follow-up is required. 

## Figures and Tables

**Table 1:** Characteristics of shift workers before and after 5-week registration period.

	N	Mean	SD		Min, Max	
Age (years)	19	40.9	11.5		26, 58	
BMI (kg/m^2^)	19	27.6	4.7			
Years in shift work	19	16.3	10.3			
	**Prior to 5-Week Shift Period**			**Following 5-Week Shift Period**		
	N	Mean	SD	N	Mean	SD
ssBP (mmHg)	19	122.9	12.0	18	122.3	8.8
sdBP (mmHg)	19	84.4	7.1	18	82.7	6.9
PWV (m/s)	19	8.0	1.5	17	8.0	1.5

BMI: Body-mass index. ssBP: Sitting systolic blood pressure. sdBP: Sitting diastolic blood pressure. PWV: Pulse wave velocity.

**Table 2:** Sleep variables assessed by diary and actigraphy.

	Diary			Actigraphy				
	N	Mean	SD	N	Mean	SD	z	*p*
NA (n)	16	1.3	0.9	17	12.9	2.7	3.52	0.0004
TST (hours)	16	6.8	0.8	17	5.8	0.8	3.31	0.0009

z/*p*-value: Wilcoxon signed-rank test. NA: Number of awakenings, TST: total sleep time.

**Table 3:** Associations between sleep and blood pressure.

	ssBP, mmHg							sdBP, mmHg							PWV						
	Crude		Adjusted					Crude		Adjusted					Crude		Adjusted				
	Coeff.	*p*-Value	Coeff.	95% CI		*p*-Value	R2	Coeff.	*p*-Value	Coeff.	95% CI		*p*-Value	R2	Coeff.	*p*-Value	Coeff.	95% CI		*p*-Value	R2
TST diary (hours)	−9.22	<0.001	−8.78	−14.30	−3.26	0.005	0.64	−7.53	<0.001	−7.51	−11.98	−3.05	0.003	0.64	−1.06	0.045	−0.24	−1.13	0.66	0.567	0.70
TST actigraphy (hours)	−2.43	0.417	−2.30	−8.27	3.67	0.418	0.28	−3.21	0.175	−3.02	−7.76	1.72	0.191	0.31	−0.01	0.989	0.08	−0.63	0.79	0.809	0.70
NA diary (n)	−1.32	0.618	−2.86	−8.73	3.01	0.307	0.32	−1.93	0.36	−2.73	−7.55	2.08	0.238	0.29	0.29	0.558	0.19	−0.53	0.91	0.566	0.70
NA actigraphy (n)	−1.73	0.031	−1.46	−3.43	0.51	0.132	0.38	−1.93	0.001	−1.95	−3.28	−0.62	0.008	0.57	−0.22	0.143	0.06	−0.18	0.30	0.593	0.71

Adjustment: Sex and age. R2: Goodness of fit.

## Data Availability

The data presented in this study are available on request from the corresponding author. The data are not publicly available due to data protection issues, as data are still being collected from the subjects.
